# Retraction: MicroRNA-935 directly targets FZD6 to inhibit the proliferation of human glioblastoma and correlate to glioma malignancy and prognosis

**DOI:** 10.3389/fonc.2026.1880372

**Published:** 2026-05-28

**Authors:** Frontiers Editorial Office

Please find the retraction statement below:

Following publication, concerns were raised regarding the integrity of the images in the published figures. The authors failed to provide a satisfactory explanation during the investigation, which was conducted in accordance with Frontiers’ policies.

This retraction was approved by the Chief Editors of Frontiers in Oncology and the Chief Executive Editor of Frontiers. The authors received a communication regarding the retraction and had a chance to respond. This communication has been recorded by the publisher.

Frontiers would like to thank the users on PubPeer for bringing the published article to our attention.

